# Intravascular lithotripsy-assisted carotid artery stenting in heavily calcified lesions: A case series

**DOI:** 10.1016/j.radcr.2025.08.087

**Published:** 2025-09-15

**Authors:** Orlando Diaz, Jochen Gerstner Saucedo, Isabel Carmona, Alan B. Lumsden, Balazs C. Lengyel

**Affiliations:** a Department of Radiology, Houston Methodist Hospital, 6565 Fannin Street, Houston, TX 77030, USA; b Advanced Imaging Lab, Department of Radiology, University of Colorado Anschutz Medical Campus, 13001 East 17th Place, Aurora, CO 80045, USA; c Universidad ICESI, School of Medicine, Calle 18 No. 122-135, Cali, Valle del Cauca, Colombia, 76003; d Department of Cardiovascular Surgery, Houston Methodist DeBakey Heart & Vascular Center, 6565 Fannin Street, Houston, TX 77030, USA; e Semmelweis Egyetem (Semmelweis University), Üllői út 26, Budapest 1085, Hungary

**Keywords:** Lithotripsy, Carotid stenosis, Off-label use, Carotid artery, Internal, Stents, Endarterectomy, Carotid

## Abstract

Intravascular lithotripsy (IVL), although considered off-label for carotid interventions, has proven to be beneficial for the treatment of heavily calcified lesions and may improve outcomes in select patients unsuitable for open repair. We present 3 consecutive cases of IVL-assisted carotid artery stenting (CAS) performed via transfemoral and transradial approaches in patients with severe internal carotid artery calcification. All patients were considered poor candidates for carotid endarterectomy (CEA). Technical success was achieved in all cases, with no neurological complications noted during the perioperative period, 30-day follow-up (with 6-month follow-up in 2 patients since 1 was lost to follow-up). All procedures were performed using distal filter protection alone without flow reversal, in contrast to most published series. IVL may broaden CAS applicability for patients with circumferential calcification and combined high anatomic/physiologic surgical risk who are not candidates for CEA. Further studies are warranted to evaluate long-term outcomes and optimal patient selection.

## Introduction

Intravascular lithotripsy (IVL) is an emerging endovascular technology that facilitates plaque modification in heavily calcified coronary arteries and peripheral arteries by using pulsatile acoustic pressure waves. However, its use in carotid interventions is still off-label and relatively unexplored [1,2]. IVL technology was originally developed from extracorporeal shockwave lithotripsy (ESWL), a urological technique that utilizes electrohydraulic lithotripsy (EHL) to fragment kidney stones [3]. Its adaptation to vascular use enables low-pressure balloon inflation to achieve vessel compliance without causing barotrauma.

Heavily calcified carotid plaque is an imaging-based concept rather than a pathognomonic finding, and currently lacks a universally accepted definition. In both clinical practice and research, `heavy' calcification is typically characterized on computed tomography angiography (CTA) or angiography by circumferential or near-circumferential involvement (exceeding 50%-100% of the vessel circumference), or by dense, bulky deposits that protrude into the lumen. These imaging patterns are associated with impaired stent expansion and an increased risk of embolic events during carotid artery stenting (CAS) [4,5].

Incidence data on heavily calcified carotid lesions and the proportion of patients deemed unsuitable for carotid endarterectomy (CEA) remain limited, largely influenced by cohort selection and imaging protocols. In a review of patients undergoing imaging for suspected or known carotid artery disease, calcification was identified in approximately 50%-60% of plaques [4]; however, severity grading was not standardized. In a CTA cohort with ≥50% ICA stenosis, calcification was present in 89% of patients, indicating that calcification is highly prevalent in moderate-to-severe carotid stenosis. In a single-center CTA-based screening study, 21% of ICAs were unsuitable for CEA due to anatomically high lesions, extending to/above C2, and 6% were ineligible for any stenting due to small vessel diameter or circumferential calcium [5].

While CEA is the preferred approach for heavily calcified plaques due to higher rates of procedural failure and embolic complications with transfemoral CAS, certain patients are poor surgical candidates. This includes those with significant cardiopulmonary comorbidities such as moderate to severe heart failure, recent myocardial infarction (<6 months), unstable angina, advanced coronary artery disease, severe pulmonary disease (eg, COPD), or renal insufficiency/dialysis dependence. Patients with prior neck surgery or irradiation may also be poor candidates due to dense scar tissue that increases dissection difficulty. In addition, challenging anatomical factors, such as high cervical lesions, high carotid bifurcation, low/common carotid bifurcation below the clavicle, or a hostile neck with extensive scarring, short or thick neck, prior cervical fusion, or severe kyphosis/immobility, can significantly increase surgical risk [6,7].

Published experience with IVL-assisted CAS/TCAR remains limited to small retrospective series and case reports. These reports are constrained by small sample sizes, highly selected high-risk cohorts, and follow-up usually ≤12 months, limiting conclusions on long-term durability and comparative outcomes [[8], [9], [10], [11], [12]]. Unlike most prior reports of IVL, which predominantly involve TCAR with flow reversal or mixed-risk lesions, our series focuses on IVL-assisted transfemoral and transradial CAS using filter protection alone in patients with circumferential ICA calcification and combined anatomic/physiologic high-risk features making CEA not feasible.

This report aims to highlight IVL’s potential role in CAS when CEA is not feasible, particularly for lesions with extreme circumferential calcification and a combination of surgical and medical risk factors that make conventional approaches challenging. By improving luminal expansion and stent apposition, IVL may provide a safer and more effective revascularization option for this complex patient population. Here, we describe our initial experience combining IVL with conventional CAS for the treatment of heavily calcified ICA lesions in patients deemed unsuitable for CEA.

## Cases presentation

We selected patients for IVL-assisted carotid artery stenting based on the presence of severe, heavily calcified stenoses that were anatomically or physiologically high-risk for carotid endarterectomy and technically challenging for conventional CAS or TCAR. All cases involved lesions with circumferential or heavily calcified that were anticipated to prevent adequate stent expansion using standard high-pressure balloon angioplasty, with the goal of using IVL to facilitate safe and effective vessel preparation.

Every procedure was performed under general anesthesia, with standard monitoring and invasive arterial pressure measurement via arterial line. Induction typically involved fentanyl 25 mcg, propofol 80 mg, rocuronium 70 mg or succinylcholine 80 mg, and dexamethasone 4 mg, with endotracheal intubation in all cases. Prophylactic medications included famotidine and ondansetron. Neuromuscular blockade was reversed with sugammadex 200 mg when appropriate.

Anticoagulation was achieved with a heparin bolus of 70 units/kg followed by an infusion of 700 units/kg, titrated to maintain an activated clotting time (ACT) between 250 and 300 seconds, using a point-of-care measurement intra-procedurally.

### Patient 1

A 70-year-old male with prior medical history of hypertension, hyperlipidemia, smoking, atrial fibrillation, coronary artery disease, and prior coronary artery bypass grafting (CABG) 13 years earlier presented for evaluation of suspected Parkinson’s disease due to recent onset of shuffling gait, flat affect, stooped posture, and resting tremor. His medical history was notable for a right-sided middle cerebral artery (MCA) stroke with resultant left-sided weakness and brachiocephalic artery stenting 7 years prior. Brain MRI showed chronic infarcts in the right MCA territory and occlusion of the left ICA. Cerebral angiography revealed 40% in-stent restenosis of the right brachiocephalic stent and an 80% calcified stenosis at the right carotid bifurcation per North American Symptomatic Carotid Endarterectomy Trial (NASCET) criteria. The left ICA and left vertebral artery (VA) were occluded.

Given the patient’s extensive circumferential calcification, contralateral carotid occlusion, high cervical lesion location, and significant comorbidities, carotid endarterectomy (CEA) was deemed high-risk for perioperative complications. Therefore, IVL-assisted carotid artery stenting (CAS) was selected to enable safe, effective plaque modification at low inflation pressures.

The procedure was performed via right radial artery access. A 7 mm x 60 mm ShockWave M5 balloon angioplasty catheter (ShockWave Medical Inc., Santa Clara, CA, USA) was used for predilatation of the stenosed segment. IVL was then performed in 3 overlapping segments, from distal to proximal, delivering 40 pulses at 2 atm and 60 pulses at 4 atm, for a total of 100 pulses. This was followed by deployment of a 10 mm x 31 mm Wallstent (Boston Scientific, Marlborough, MA, USA). Poststent dilatation was performed with a 10 mm x 20 mm Sterling balloon catheter (Boston Scientific, Marlborough, MA, USA). A SpiderFX embolic protection device (Medtronic, Dublin, Ireland) was used during the procedure.

**Fig. 1:**
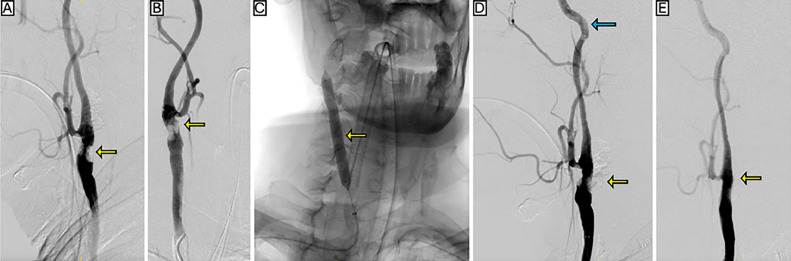
(A,B) Diagnostic angiography demonstrating a heavily calcified plaque at the distal common carotid artery (CCA), indicated by yellow arrows, resulting in 80% stenosis by NASCET criteria. (C) Intravascular lithotripsy using a 7 mm x 60 mm ShockWave M5 balloon at the right carotid bifurcation (yellow arrow). (D) Angiographic appearance immediately after lithotripsy, showing persistent narrowing at the plaque site (yellow arrow) with the distal embolic protection device positioned beyond the lesion in the ICA (blue arrow). (E**)** Completion angiography after deployment of a 10 mm x 31 mm stent, showing resolution of the stenosis (yellow arrow).

Completion angiography confirmed successful stent deployment with no residual stenosis or complications. The filter basket was found empty upon removal. The patient’s postoperative course was uneventful. He was maintained on single antiplatelet therapy (clopidogrel 75 mg daily) and apixaban 5 mg twice daily for atrial fibrillation, with no additional pharmacologic therapy. No neurological symptoms were noted during the perioperative period or at the 30-day follow-up (Fig. 1). The patient was subsequently lost to follow-up and was therefore unable to complete the planned 6-month surveillance

### Patient 2

A 71-year-old male patient with a history of hypertension, hyperlipidemia, and type II diabetes mellitus was found to have an asymptomatic right ICA stenosis during preoperative evaluation for a planned CABG. He underwent heart surgery without complications. After recovery, he was referred for further evaluation of the known carotid stenosis. Duplex ultrasound (US) was limited due to acoustic shadowing from a heavily calcified plaque. A cerebral angiogram confirmed a 95% stenosis of the right ICA according to NASCET criteria, with a normal left ICA and left VA, and a hypoplastic right VA.

Given the patient’s severe circumferential calcification, hypoplastic contralateral VA, and recent major cardiac surgery, carotid endarterectomy (CEA) was considered high-risk for perioperative complications. Initial treatment with conventional transfemoral CAS was attempted; however, the embolic protection device could not be advanced across the lesion, and despite multiple balloon angioplasty inflations reducing the stenosis from 95% to 70%, the stent could not be delivered or positioned. The degree and rigidity of the calcification were deemed the primary limiting factors for successful stent deployment. Therefore, the decision was made to re-intervene the following day using IVL to facilitate safe and effective plaque modification at low pressures, enabling definitive CAS.

**Fig. 2:**
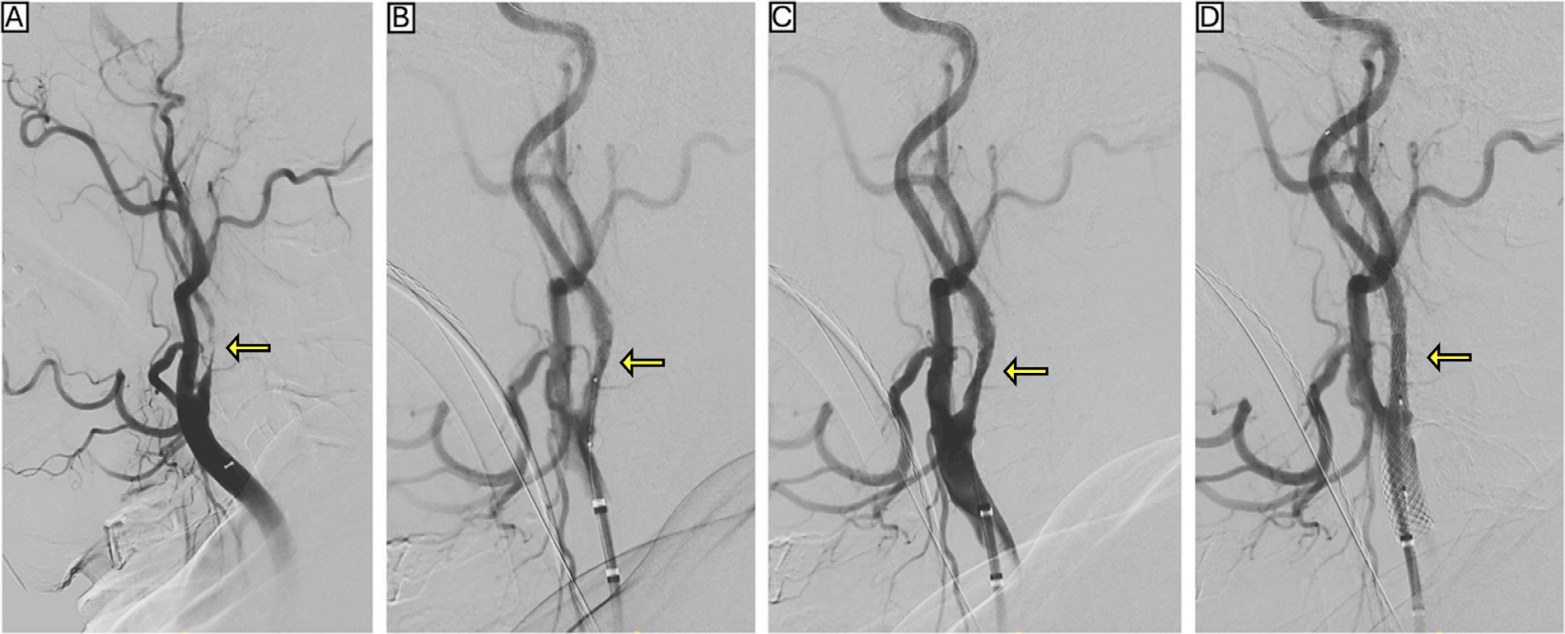
(A) Diagnostic angiography showing severe, calcified subtotal stenosis of the right internal carotid artery (ICA), indicated by the yellow arrow. (B–C) Angiographic images during intravascular lithotripsy using a 4 mm x 12 mm ShockWave M5 balloon, with the treated segment highlighted by yellow arrows. The embolic protection device is not visualized as it is positioned distally and (D) Completion angiography after deployment of a 10 mm x 31 mm stent, demonstrating restored luminal patency without complication (yellow arrow).

On the second attempt, via right femoral access, after placement of a SpiderFX embolic protection device (Medtronic, Dublin, Ireland) in the distal ICA, a 4 mm x 12 mm ShockWave C2+ balloon angioplasty catheter (ShockWave Medical Inc., Santa Clara, CA, USA) was advanced across the lesion. Lithotripsy was delivered in multiple segments from distal to proximal, starting with 30 pulses at 2 atm and 30 pulses at 4 atm, followed by proximal treatment with 30 pulses at 2 atm and 30 pulses at 4 atm, for a total of 120 pulses. A 10 mm x 31 mm Wallstent (Boston Scientific, Marlborough, MA, USA) was subsequently deployed and postdilated with a 5.5 mm x 20 mm Sterling balloon catheter (Boston Scientific, Marlborough, MA, USA). Completion angiography confirmed resolution of the stenosis without complications. Inspection of the embolic protection device showed no debris. The patient continued on dual antiplatelet therapy with aspirin 100 mg daily and clopidogrel 75 mg daily, and was discharged on postoperative day 1 without any new neurological symptoms. No additional pharmacologic therapy was administered, and the patient remained symptom-free at the 30-day follow-up (Fig. 2) and at the 6-month follow-up visit. No imaging was performed at 6 months, as the patient was asymptomatic and no further follow-up was deemed necessary.

### Patient 3

An 84-year-old male patient with a history of hypertension, atrial fibrillation, prior CABG, and chronic left foot drop was evaluated for carotid stenosis after having a stroke 2 months prior (part of stroke workup). Initial carotid duplex US was inconclusive due to acoustic shadowing from extensive calcification at both carotid bifurcations. Subsequent CTA demonstrated chronic occlusion of the right ICA, 80% stenosis of the left ICA per NASCET criteria, and patent bilateral vertebral arteries.

Given the patient’s advanced age, significant cardiopulmonary comorbidities, and high cervical location of the left ICA lesion, CEA was considered high-risk for perioperative complications. The lesion’s heavy circumferential calcification also increased the technical difficulty of CEA and the risk of incomplete plaque removal. These factors, in accordance with current vascular surgery guidelines, made the patient a poor candidate for surgical revascularization. Consequently, CAS was pursued to provide a less invasive revascularization strategy with the potential for reduced perioperative morbidity, while IVL was incorporated to facilitate safe plaque modification at low inflation pressures.

**Fig. 3:**
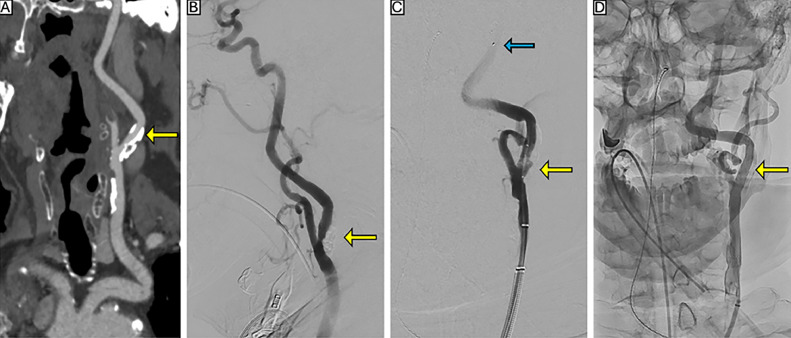
(A) CTA reconstruction demonstrating a heavily calcified plaque at the origin of the left internal carotid artery (ICA), causing approximately 80% stenosis by NASCET criteria (yellow arrow). (B**)** Digital subtraction angiography confirming the location and severity of the calcified lesion (yellow arrow). (C**)** Intravascular lithotripsy performed using a 4 mm × 12 mm ShockWave C2+ balloon (yellow arrow) with the distal embolic protection device positioned beyond the lesion in the ICA (blue arrow) and (D) Completion angiography following deployment of an 8 mm x 29 mm stent, demonstrating successful luminal restoration (yellow arrow).

Via left femoral arterial access, the lesion was successfully crossed, and a filter-wire embolic protection device (SpiderFX, Medtronic, Dublin, Ireland) was deployed. A 4 mm x 12 mm ShockWave C2+ balloon angioplasty catheter (ShockWave Medical Inc., Santa Clara, CA, USA) was used for plaque modification, delivering 20 pulses at 2 atm followed by 20 at 3 atm distally, and then 20 pulses at 2 atm and 20 at 4 atm proximally, for a total of 80 pulses. An 8 mm x 29 mm Wallstent (Boston Scientific, Marlborough, MA, USA) was then deployed and postdilated using a 6 mm x 20 mm Sterling balloon catheter (Boston Scientific, Marlborough, MA, USA). Upon retrieval, no debris was noted in the embolic protection basket. The patient remained symptom-free and was discharged on postoperative day 1 with ticagrelor 90 mg twice daily (selected due to clopidogrel resistance confirmed by P2Y12 testing), apixaban 2.5 mg twice daily, and rosuvastatin 20 mg daily. No other pharmacologic agents were used. The patient remained asymptomatic at both the 30-day follow-up (Fig. 3) and the 6-month visit. No imaging was performed at 6 months, as the patient was clinically stable and further follow-up was not deemed necessary.

An overview of patients’ clinical characteristics, procedural details, and outcomes is shown in Table 1.

**Table 1:** Summary of patient characteristics, procedural details, and outcomes for IVL-assisted carotid artery stenting.

Variable	Patient 1	Patient 2	Patient 3
Age	70	71	84
Gender	Male	Male	Male
Comorbidities	HTN HLD Smoker Afib CAD Prior CABG Prior MCA stroke	HTN HLD DM2 Recent CABG	HTN Afib Prior CABG Chronic foot drop
Reason not suitable for CEA	Contralateral ICA occlusion Contralateral VA occlusion Circumferential calcification Cardiopulmonary comorbidities	Severe calcification Hypoplastic contralateral VA Recent major cardiac surgery	Advanced age High cervical lesion Severe calcification Significant comorbidities
Lesion location	Right carotid bifurcation	Right ICA	Left ICA
Degree of stenosis per NASCET (%)	80	95	80
Access route	Right radial	Right femoral	Left femoral
IVL balloon size	7 mm × 60 mm	4 mm × 12 mm	4 mm × 12 mm
Total pulses	100 pulses (40 at 2 atm, 60 at 4 atm)	120 pulses (60 at 2 atm, 60 at 4 atm)	80 pulses (40 at 2 atm, 40 at 4 atm)
Stent type/Size	10 mm × 31 mm	10 mm × 31 mm	8 mm × 29 mm
Medication at discharge	Clopidogrel 75mg daily Apixaban 5mg BID	Clopidogrel 75mg daily Aspirin 100mg daily	Ticagrelor 90 mg BID Apixaban 2.5 mg BID Rosuvastatin 20 mg daily
Outcome	No complications Symptom-free at 30 days No 6-month follow-up	No complications Symptom-free at 30 days and 6 months	No complications Symptom-free at 30 days and 6 months

Afib, atrial fibrillation; ATM, atmospheres; BID, twice daily; CAD, coronary artery disease; CABG, coronary artery bypass grafting; CTA, computed tomography angiography; CEA, carotid endarterectomy; CAS, carotid artery stenting; DM2, type 2 diabetes mellitus; HTN, hypertension; HLD, hyperlipidemia; ICA, internal carotid artery; IVL, intravascular lithotripsy; MCA, middle cerebral artery; NASCET, North American Symptomatic Carotid Endarterectomy Trial; US, ultrasound; VA, vertebral artery.

## Discussion

IVL is a promising alternative for the treatment of heavily calcified atherosclerotic plaques. IVL generates controlled microfractures in the calcium located within the intimal and medial layers of the vessel wall by transmitting circumferential acoustic shockwaves via a specialized balloon catheter, therefore enhancing vascular compliance and facilitating luminal gain at reduced inflation pressures [3]. Unlike conventional high-pressure noncompliant balloons, which rely solely on radial force and may fail to fracture concentric calcium, IVL achieves plaque modification without excessive barotrauma, reducing the risk of vessel injury (dissection or perforation) and lowering embolic potential. This method uses less pressure and reduces the particle debris associated with atherectomy [7,8,10].

Other methods used before stenting such as rotational or orbital atherectomy, typically under flow-reversal protection, have seen limited adoption in the ICA largely because they carry a higher risk of distal microembolization [13,14]. In contrast, IVL modifies calcified plaque without tissue ablation, a mechanism already widely adopted in coronary and peripheral artery interventions, making it an appealing option for heavily calcified carotid lesions in appropriately selected patients [2].

Despite these advantages, IVL use in the carotid arteries remains off-label, and current evidence consists mainly of isolated case reports and small series of patients with heavily or circumferentially calcified lesions that would otherwise not be candidates for CAS or TCAR. In a multicenter study by Giannopoulos et al., IVL-assisted CAS in 21 high-risk patients achieved technical success in all instances, with <30% residual stenosis and no IVL-related complications [10]. Similar results were seen by DiLosa et al. [9] in a TCAR group, with a 1.7% periprocedural stroke rate and high patency at short-term follow-up. Both studies relied on flow reversal or dual protection (proximal balloon plus a distal filter), whereas our series used distal filter protection alone. Proximal occlusion itself arrests flow and may cause intolerance in patients with poor collateral circulation; additional device steps can prolong ischemic time and procedural complexity [6]. Our series adds to this evidence by showing safe IVL-enabled expansion using distal filter protection alone, which may be relevant for centers without TCAR capability or in cases where a simpler transfemoral or transradial approach is preferred.

In all 3 cases, IVL-assisted CAS achieved 100% technical success without peri-procedural complications or new neurological deficits. The treated lesions featured circumferential ICA-origin calcification and small vessel diameters, making stent expansion without IVL unfeasible and an anatomically more complex procedure than those in earlier studies [5]. All patients presented with combined anatomic and physiologic high-risk factors, such as prior neck surgery, cardiopulmonary comorbidities, and challenging arch anatomy, rendering CEA unsuitable. Notably, in 1 case, luminal expansion was achieved at relatively low balloon pressures (2–3 atm), after failure with a conventional high-pressure balloon highlighting IVL’s ability to modify heavily calcified plaques effectively.

A limiting factor in IVL’s broader use for carotid interventions is the restricted range of balloon sizes currently available. Coronary IVL balloons tend to be short and narrow, while peripheral IVL balloons are longer and designed for larger vessel diameters, neither format is ideal for the carotid anatomy. In our series, we adapted available devices and each lesion was treated in overlapping segments from distal to proximal to ensure full plaque coverage.

A key procedural distinction is that, unlike traditional balloon angioplasty which typically involves a single, brief, high-pressure inflation to reduce embolic risk and baroreceptor-mediated hypotension, IVL requires multiple cycles of prolonged (∼30 s) low-pressure inflation. This slower, staged approach allows acoustic shockwaves to modify the plaque but could theoretically provoke hypotension via baroreceptor activation [15]. However, in our series, performed under general anesthesia without neuromonitoring, no hemodynamic instability occurred.

While these early results are encouraging, they must be interpreted within the context of important limitations. Our follow-up was limited to 30 days in all patients and 6 months in 2 cases, similar to many prior reports, and does not allow conclusions regarding longer-term patency, restenosis, or stroke prevention. The series is also small, reflecting the rarity of this highly selected patient subset, and lacks a comparator group. Nonetheless, our experience suggests that IVL can be safely integrated into CAS for patients with complex anatomy and heavy calcification, potentially expanding treatment options for no-option patients, particularly in centers without TCAR capability or where a simpler transfemoral or transradial strategy is preferred.

Further studies with a larger number of patients, longer follow-up and comparative designs are warranted to provide insights into the neurological outcomes, optimal device selection for IVL-assisted carotid interventions and long-term results of the procedure.

## Conclusion

We reported 2 cases of successful transfemoral, and 1 transradial carotid artery stenting using IVL for predilatation without any major complications. All procedures were technically successful and free of major complications. These results suggest that IVL may facilitate safe and effective stent expansion in anatomically and physiologically high-risk patients who are poor candidates for CEA and in whom conventional CAS is technically challenging. By enabling treatment at low inflation pressures and with simplified protection strategies, IVL could broaden the applicability of CAS, particularly in centers without TCAR capability or in patients requiring alternative access routes. Larger studies with extended follow-up are warranted to define its long-term efficacy and safety profile.

## Patient consent

Written informed consent was obtained from all 3 patients for publication of their cases and accompanying images.
